# Unilateral Purple Urine Bag Syndrome in an Elderly Man with Nephrostomy

**DOI:** 10.7759/cureus.5435

**Published:** 2019-08-20

**Authors:** Behzad Amoozgar, Pavan Garala, Vasilios N Velmahos, Bhavana Rebba, Shuvendu Sen

**Affiliations:** 1 Internal Medicine, Jersey Shore University Medical Center (Perth Amboy), Perth Amboy, USA; 2 Internal Medicine / Infectious Disease, Hackensack Meridian Health JFK Medical Center, Edison, USA

**Keywords:** purple urine bag syndrome, urinary tract infection, catheter malfunctioning, nephrostomy tube

## Abstract

Purple urine bag syndrome, or PUBS, is a manifestation of a complicated urinary tract infection. Organisms such as Escherichia coli (E. coli) and Enterococcus can reside in urinary catheters and exhibit the purple color detected in this phenomenon. Risk factors described for this syndrome include the use of plastic urinary catheterization, the malfunctioning of the catheter, and long-term institutionalization. This disorder could be the earliest presentation of a urinary catheter flaw and requires immediate intervention and revision. In our case, a male resident of nursing home presented with urosepsis and appropriate antibiotics were initiated. Computed tomography (CT) urogram was done and showed left kidney hydronephrosis and bilateral staghorn calculi. To address the source of infection, a nephroureteral drain was placed in both kidneys. A few days after the initiation of treatment and urological intervention, urine on the left side became purple. The urologist re-evaluated the nephroureteral drainage tubes and replaced them. The purple color in the urine resolved later. In our case, PUBS was the earliest sign of urinary drainage malfunctioning and required early intervention and treatment.

## Introduction

Purple urine bag syndrome (PUBS) is a benign manifestation of a urinary tract infection. Both Escherichia coli (E. coli) and the Enterococcus family can cause PUBS. The causative bacteria produce sulphatases and phosphatases, which through tryptophan metabolism results in the formation of indigo and indirubin pigments that, in combination, exhibit purple color. One of the reasons for the occurrence of PUBS is the increased concentration of bacteria in the urine as the result of urinary outflow obstruction. Nephrostomy tube malfunctioning can result in urinary obstruction and facilitate the environment for bacteria overgrowth and PUBS manifestation [1-2].

Here, we present a rare case of PUBS that manifested as unilateral purple urine collection in a patient with a nephrostomy tube and suprapubic catheter.

## Case presentation

This was a 61-year-old male, chronic nursing home resident who presented to the emergency room (ER) with fever (102.7°F) and hypotension. The patient had a past medical history of anoxic brain injury owing to cardiac arrest. He also had a past urologic history of a chronic suprapubic catheter due to a neurologic bladder, left percutaneous nephrostomy due to obstructive uropathy, and left-sided nephroureteral stent because of an obstructing mid-ureteral stone. In addition, the patient had a history of multiple admissions due to urosepsis.

On examination, the patient was found to be lethargic and looked dehydrated. His labs during admission were: white blood cell (WBC) count 31.6 10^3^/µL, blood urea nitrogen (BUN) 23 mg/dL, and creatinine 2.3 mg/dL. Additionally, the procalcitonin level came back to 140 ng/mL. Urine showed a high number of WBCs, red blood cells (RBCs), and bacteria but the level for nitrite was in the normal range.

**Figure 1:**
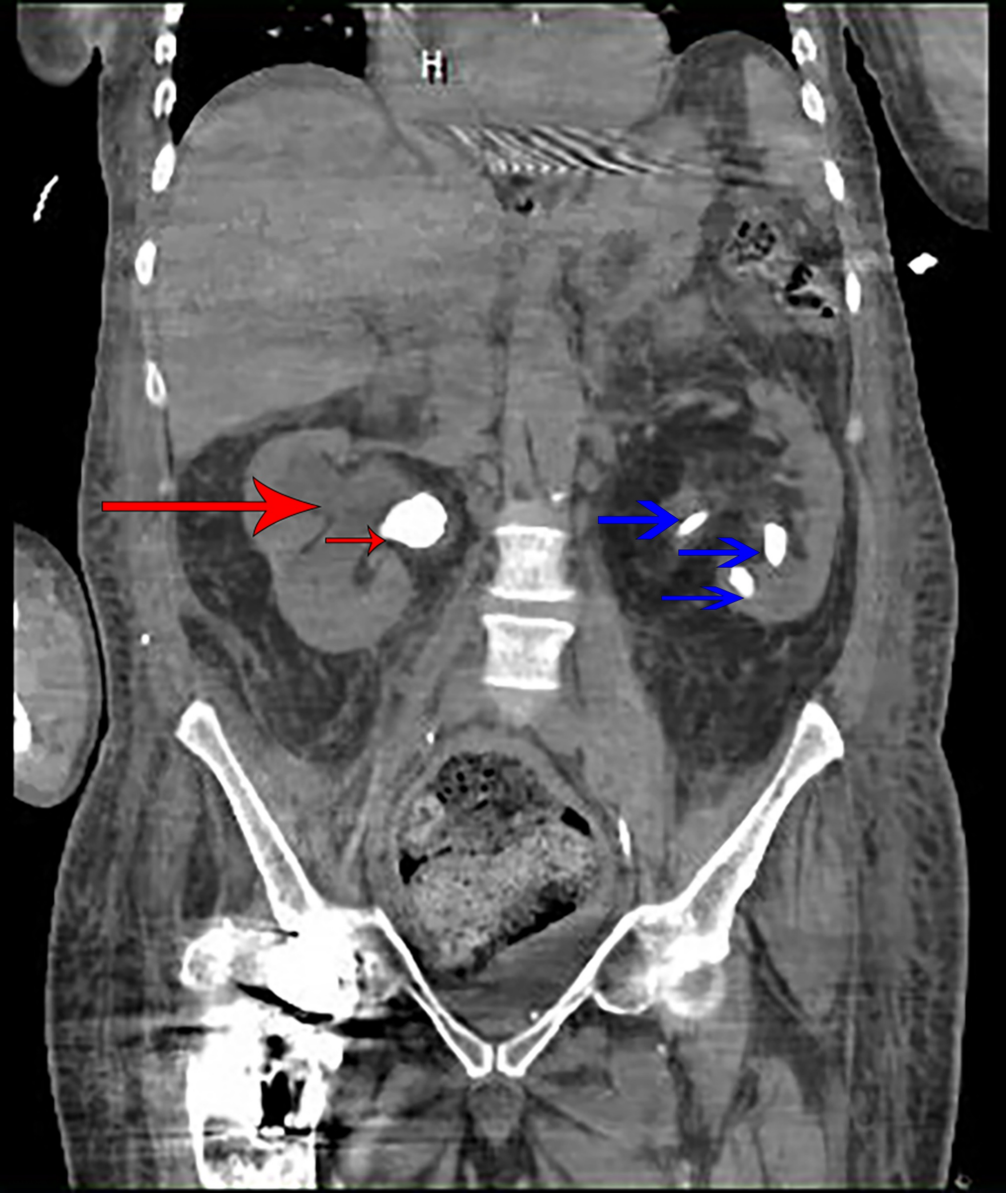
Computed tomography urogram shows moderate right-sided hydronephrosis (large red arrow), one of the staghorn calculi in the right kidney (small red arrow) and three staghorn calculi within the left kidney (three blue arrows)

The patient did not respond to fluid resuscitation, was diagnosed with septic shock, and was subsequently admitted to the intensive care unit (ICU) with a diagnosis of septic shock. Infectious disease and urology services were consulted and empirical treatment was initiated with meropenem and vancomycin. Blood and urine cultures were collected and the urologist replaced the old left nephrostomy catheter. Computed tomography (CT) urogram showed moderate right-sided hydronephrosis. Two staghorn calculi were detected in the right kidney and ureteropelvic junction (Figure 1). Moreover, multiple staghorn calculi were seen in the left kidney (Figure 1).

**Figure 2:**
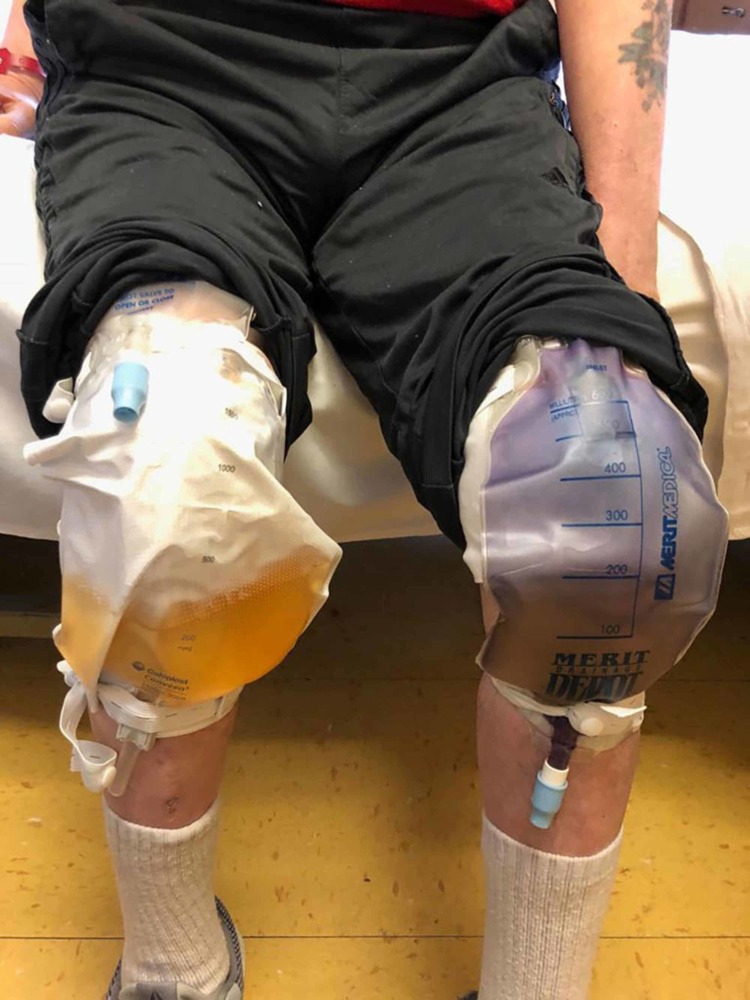
Comparison between the suprapubic catheter (yellow/left) and the nephrostomy catheter (purple/right)

Urine culture from the suprapubic catheter came positive for E. coli, Proteus mirabilis, and Pseudomonas aeruginosa. Urine culture from the left nephrostomy tube was negative. Furthermore, blood culture came back positive for Enterococcus fecalis. Awaiting the second blood culture, infectious disease (ID) changed the antibiotic regimen from meropenem to piperacillin-tazobactam and vancomycin (empirical coverage for gam positive bacteria originated from urinary tubes). To further address the source of infection and allow urine flow from the kidney, a nephroureteral drainage catheter/stent was placed in both kidneys six days after admission (exchanged with the nephrostomy tube in the left kidney). Interestingly, eight days after the left nephrostomy tube replacement, the urine color became purple while urine from the suprapubic catheter remained yellow (Figure 2).

**Figure 3:**
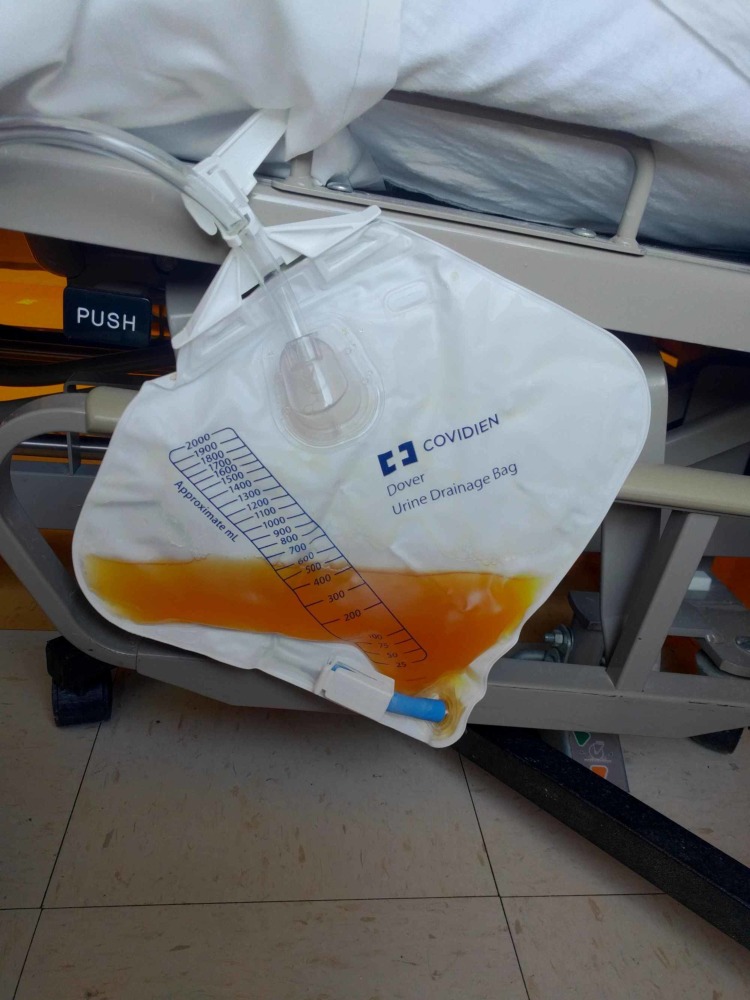
After nephroureteral catheter replacement

The patient's condition improved, and he was discharged to a nursing home. Purple urine remained despite appropriate coverage. Two weeks after returning to the nursing home, the urologist re-evaluated the patient and decided that the nephroureteral catheters/drainage on both sides were malfunctioning. Hence, both nephroureteral catheters were replaced and a few days later, the patient’s urine turned yellow again (Figure 3).

## Discussion

The rate of urinary tract infection (UTI) increases with age [3-5]. For chronic catheter users and residents of long-term care facilities, the rates of UTI are even higher [6-7].

PUBS is a rare phenomenon and was first reported in 1978 [8]. PUBS is the result of UTI with bacteria that metabolize the products of tryptophan and produce the oxidized indole derivatives, indigo and indirubin. Two latter products react with catheter tubing and manifest as a purple hue. Interaction between the urine bag plastic and the aforementioned pigments, as well as a high bacterial load, are important in precipitating PUBS [9-12]. Among the bacterial aetiologies, Proteus mirabilis, Escherichia coli, Enterococcus species, Morganella morganii, Pseudomonas aeruginosa, Providencia stuartii, Providencia rettgeri, Klebsiella pneumoniae, Citrobacter species, and group B Streptococci are the most common pathogens contributing to PUBS [13].

Female gender, use of plastic urinary catheterization (8.3% to 16.7% in long-term urinary catheterized patients), alkaline urine, constipation, and institutionalization are the most common risk factors associated with PUBS [13].

In this case, PUBS manifested during the administration of an appropriate antibiotic regimen. The repeat urine culture was negative and PUBS resolved after the second revision and replacement of the left nephrostomy tube. We suspect that E. coli resulting in PUBS in the left nephrostomy bag could produce biofilms and hence remain active for a longer period of time after the initiation of the antibiotic regimen. The biofilm is among the pathogen’s key virulent factors; it allows the pathogen to escape host defense mechanisms and enhances antimicrobial resistance due to slow penetration (both antibiotics and white blood cells) and alteration of the microenvironment [14].

PUBS may become potential guidance for the evaluation of catheter function, specifically in an asymptomatic patient while receiving antibiotics.

## Conclusions

PUBS is a rare manifestation of UTI and is relatively benign in its clinical course. Longstanding catheterization and residing in long-term care facilities are common risk factors. Physicians should focus on the prevention of PUBS by shortening the duration of catheterization or elimination of catheter blockage. This phenomenon may alarm clinician in regards to urinary catheter patency or the characteristics of the bacteria residing at the site of the urinary catheter.
